# Prognostic value of isocitrate dehydrogenase 1, *O*^6^-methylguanine-DNA methyltransferase promoter methylation, and 1p19q co-deletion in Japanese malignant glioma patients

**DOI:** 10.1186/1477-7819-11-284

**Published:** 2013-10-25

**Authors:** Yoshinobu Takahashi, Hideo Nakamura, Keishi Makino, Takuichiro Hide, Daisuke Muta, Hajime Kamada, Jun-ichi Kuratsu

**Affiliations:** 1 Department of Neurosurgery, Graduate School of Medical Science, Kumamoto University, 7-5, Inada, Obihiro, Hokkaido 080-0039, Japan; 2 Department of Neurosurgery, Hokuto Hospital, 7-5, Inada, Obihiro, Hokkaido 080-0039, Japan

**Keywords:** IDH1, MGMT methylation, 1p19q co-deletion, Malignant glioma, Prognosis

## Abstract

**Background:**

To determine the prognostic value of isocitrate dehydrogenase 1 (*IDH1*) mutation, *O*^6^-methylguanine-DNA methyltransferase (*MGMT*) promoter methylation, and 1p/19q co-deletion in Japanese patients with malignant gliomas.

**Methods:**

We studied 267 malignant gliomas, which included 171 glioblastomas (GBMs), 40 anaplastic astrocytomas (AAs), 30 anaplastic oligodendrogliomas (AOs), and 26 anaplastic oligoastrocytomas (AOAs). These malignant gliomas were divided into 2 groups (Group 1: GBM + AA, Group 2: AO + AOA) according to the presence of the oligodendroglioma component. We examined *IDH1* mutation and *MGMT* promoter methylation in each group by direct sequencing and methylation-specific PCR, respectively. We further examined 1p/19q co-deletion in Group 2 by fluorescence in situ hybridization. Survival between groups was compared by Kaplan–Meier analysis.

**Results:**

In Group 1, patients with *IDH1* mutations exhibited a significantly longer survival time than patients with wild-type *IDH1*. However, no significant difference was observed in Group 2, although patients with *IDH1* mutations tended to show prolonged survival. For both Group 1 and Group 2, patients with *MGMT* methylation survived longer than those without this methylation. Further, patients with 1p/19q co-deletion showed significantly better outcome in Group 2.

**Conclusions:**

Our study confirms the utility of *IDH1* mutations and *MGMT* methylation in predicting the prognosis of Group 1 patients (GBM + AA) and demonstrated that *IDH1* mutations may serve as a more reliable prognostic factor for such patients. We also showed that *MGMT* methylation and 1p/19q co-deletion rather than *IDH1* mutations were prognostic factors for Group 2 patients (AOA + AO). Our study suggests that patients survive longer if they have *IDH1* mutations and undergo total resection. Further, irrespective of *MGMT* promoter methylation status, the prognosis of glioma patients can be improved if total resection is performed. Moreover, our study includes the largest number of Japanese patients with malignant gliomas that has been analyzed for these three markers. We believe that our findings will increase the awareness of oncologists in Japan of the value of these markers for predicting prognosis and designing appropriate therapeutic strategies for treating this highly fatal disease.

## Background

Malignant gliomas are the most common type of primary brain tumor. They are classified on the basis of the World Health Organization (WHO) grading system. Pathological diagnosis helps ascertain the biology and behavior of brain tumors. The most commonly used consensus approach for the diagnosis of malignant gliomas is to classify the tumors as astrocytic tumors, that is, anaplastic astrocytoma (AA),glioblastoma (GBM),anaplastic oligodendroglioma (AO), and anaplastic oligoastrocytoma (AOA). An accurate distinction between the different types of malignant gliomas is important for deciding the prognosis and therapeutic approaches. Thus far, histopathological examination is the gold standard for the typing and grading of gliomas. However, this method is associated with significant inter-observer variability. Furthermore, the clinical behavior of individual tumors having specific pathology might differ substantially. Thus, additional markers are needed for refined and more objective glioma classification, better prediction of prognosis, and tailored therapeutic decision-making. At present, clinical factors such as age, Karnofsky performance status (KPS), and resection rate are primarily used to predict the prognosis.

Unlike the classical molecular markers for gliomas - *p53* and epidermal growth factor receptor (*EGFR*) status - the clinical significance of which has remained controversial, at least three important molecular markers with clinical implications have now been identified. These are 1p/19q co-deletion, *O*^6^-methylguanine methyltransferase (*MGMT*) promoter methylation, and isocitrate dehydrogenase-1 (*IDH1*) mutations.

Chromosome 1p/19q co-deletion was first reported in oligodendroglial tumors in 1994 [1]. Cairncross *et al*. reported chemosensitivity in patients with AOs harboring deletion of 1p, particularly co-deletion of 1p and 19q [2]. Almost 85% of low-grade oligodendrogliomas and 65% of AOs harbor 1p/19q co-deletion [3]. The potential role of 1p/19q loss in therapeutic decision-making in AOs has been analyzed in large studies. The 1p/19q deletions were incorporated into three major therapeutic trials in patients with AO. All the trials confirmed the prognostic and possible predictive role of this biomarker at initial therapy [4-6].

*MGMT* promoter methylation is the only potentially predictive marker, especially for alkylating agent chemotherapy in glioblastoma. At present, temozolomide (TMZ) is mainly used for the treatment of malignant gliomas [7], and many clinical studies on TMZ have been performed. TMZ is a DNA-methylating agent and exerts its cytotoxicity by adding a methyl group to the *O*^6^ position of guanine residues on DNA. This induces DNA mismatch, DNA double-stand breaks, and apoptosis in proliferating cells [8]. *MGMT*, a DNA repair enzyme, is known to induce resistance to chemotherapy in some patients with malignant gliomas. In a tumor with a hypermethylated *MGMT* promoter, *MGMT* expression is reduced and cytotoxicity of alkylating agents is enhanced. Stupp *et al*. suggested that the combination of TMZ with radiotherapy could be used as the initial standard treatment for GBM [9]; they also investigated whether the state of *MGMT* activity could be a prognostic factor. Cancer-specific DNA methylation changes are hallmarks of human cancers, with global DNA hypomethylation often seen concomitantly with hypermethylation of CpG islands [10]. A CpG island methylator phenotype (CIMP) is regarded as cancer-specific CpG island hypermethylation of a subset of genes in some tumors [11]. In GBM, glioma-CIMP status (G-CIMP) has been shown to be a significant predictor of improved patient survival [12]. Collectively, these different sets of observations suggest that the level of *MGMT* promoter methylation, serving as a prognostic factor, may reflect an aspect of the global DNA methylation status in GBM.

In 2008, Volgelstein *et al*. conducted a comprehensive sequence analysis in 22 patients with GBM and identified *IDH1* mutation as a new driver mutation [13]. In another analysis, they detected *IDH1* mutations in 18 (12%) of 149 patients with GBM. Clinically, patients with *IDH1* mutations are characterized by the occurrence of secondary GBM and early disease onset [14,15]. A large-scale study revealed *IDH1* mutations in 50% to 80% of patients with grade 2 astrocytoma, oligodendroglioma, or secondary GBM; however, *IDH1* mutations were rare in patients with primary GBM [6,16-24]. Thus, *IDH1* mutations may be considered new molecular diagnostic markers. In addition, recent studies showed that patients with *IDH1* mutations had a better outcome than those with wild-type *IDH1*[6,16-24]. The biological function of *IDH1* mutations has not yet been completely understood. Wild-type *IDH1* oxidizes isocitrate to α-ketoglutarate (α-KG) and reduces nicotinamide adenine dinucleotide phosphate (NADP) to NAPD-oxidase (NADPH) [25]. Mutated *IDH1* reduces the activity of NADPH, which is required for cellular defense against oxidative stress, leading to tumorigenesis because of oxidative DNA damage [26]. Furthermore, this mutation results in a new function of *IDH1* leading to the conversion of α-KG to 2-hydroxyglutarate (2HG), which promotes the accumulation of hypoxia-inducible factor (HIF)1α, leading to vascular endothelial growth factor signaling-mediated tumorigenesis *in vitro*[27]. However, Metellus *et al*. question the actual relationship between *IDH* mutation status and *in vivo* hypoxic biomarkers [28]. Also Chowdhury *et al*. showed that 2HG inhibits 2-oxoglutarate (2OG)-dependent oxygenases with varying potencies and indicated that candidate oncogenic pathways in *IDH*-associated malignancy should include those that are regulated by other 2OG oxygenases than HIF hydroxylases [29]. Despite its obvious association with tumorigenesis, the relationship between *IDH1* mutation and good prognosis for malignant glioma is yet unknown.

We evaluated the significance of these markers, that is, 1p/19q co-deletion, *MGMT* promoter methylation, and *IDH1* mutations, in malignant glioma. The objective of the present study was to confirm the difference in the prognostic impacts of *MGMT* methylation status and *IDH1* mutation and 1p/19q co-deletion in patients with GBM and AA and those with AO and AOA, respectively.

## Methods

In this study, patients with malignant glioma were divided into two groups according to the presence of the oligodendroglioma component. Groups 1 and 2 consisted of patients with GBM and AA and those with AO and AOA, respectively.

### Patient and tissue specimens

Between 1996 and 2009, 267 patients with malignant glioma (30 with AO, 26 with AOA, 40 with AA, 159 with primary GBM and 12 with secondary GBM) treated at Kumamoto University Hospital were included in this study. Tumor specimens were obtained by surgical resection (including biopsy), quick-frozen in liquid nitrogen, and maintained at -80°C until use. The patients and/or their legal guardians provided written informed consent for use of the specimens. Formalin-fixed, paraffin-embedded specimens were pathologically examined. Each specimen was classified by the local neuropathologists according to the WHO criteria. The tumor type *IDH1* mutational status, *MGMT* methylation status, age and gender distribution, Karnofsky performance status (KPS) score, and median survival time are shown in Table 1.

**Table 1:** Patients and characteristics

	**Histologic subtype**			
**Characteristic**	**AO (n = 30)**	**AOA (n = 26)**	**AA (n = 40)**	**GBM (n = 171)**
**Gender**				
Male/female ratio	0.76	1.36	1.22	1.59
Male, n	13	15	22	105
Female, n	17	11	18	66
**Age, years**				
Median	45.0	49.5	45.5	61.0
Range	16 to 77	30 to 65	10 to 72	3 to 81
**Karnofsky performance status**				
Median	100	100	90	90
Range	40 to 100	70 to 100	40 to 100	20 to 100
**Surgery**				
Total removal, n	22	13	8	74
Partial removal, n	7	12	21	73
Biopsy, n	1	1	11	24
***IDH1 *** **mutation, n**	20(66.7%)	12(46.2%)	12(30.0%)	12(7.0%)
***MGMT *** **promoter methylation, n**	24(80.0%)	19(73.1%)	18(45.0%)	73(42.7%)
**1p/19q co-deletion, n**	18(60.0%)	11(42.3%)		
**Survival, months, median**	70.5	80.0	40.0	14.0

AO, anaplastic oligodendroglioma; AOA, anaplastic oligoastrocytoma; AA, anaplastic astrocytoma; GBM, glioblastoma; n, number of patients.

### Direct DNA sequencing of *IDH1* mutations

Genomic DNA was isolated from the surgical specimens using the Qiagen kit (Qiagen, Valencia, CA, USA). The PCR primers for genomic region corresponding to *IDH1* exon 4 that encodes codon R132 were as follows: *IDH1* sense (5′-AAACAAATGTGGAAATCACC-3′) and *IDH1* antisense (5′-TGCCAACATGACTTACTTGA-3′). The PCR conditions were 94° for 5 minutes; 36 cycles of 94°C for 30 s, 55°C for 30 s, and 72°C for 1 minute; and extension at 72°C for 5 minutes. The PCR was performed using Ex-Taq HS DNA Polymerase (Takara Bio, Shiga, Japan). The PCR products were purified using QIAquick PCR Purification Kit (Qiagen) according to the manufacturer’s instructions. Sequencing reactions were performed using previous primers and a Big Dye Terminator Cycle Sequencing Kit (Applied Biosystems, Life Technologies, Carsbad, CA, USA) on an ABI377 automated sequencer (Applied Biosystems).

### Methylation-specific PCR for *MGMT* promoter

*MGMT* methylation was detected using methylation-specific PCR (MSP). Genomic DNA from each sample (2 μg) was treated with sodium bisulfite using the Epitect Bisulfite Kit (Qiagen Valencia, CA). The primer sequences for the unmethylated reaction were 5′-TTTGTGTTTTGATGTTTGTAGGTTTTTGT-3′ (forward) and 5′-AACTCCACACTCTTCCAAAAACAAAACA-3′ (reverse), and those for the methylated reaction were 5′-TTTCGACGTTCGTAGGTTTTCGC-3′ (forward) and 5′-GCACTCTTCCGAAAACGAAACG-3′ (reverse). The PCR conditions were as follows: 95° for 5 minutes; 34 cycles of 95° for 30 s, 61° for 30 s, 72° for 30 s; and extension at 72° for 4 minutes. Amplified products were separated on 3% agarose gels, stained with ethidium bromide, and visualized under UV illumination.

### 1p/19q co-deletion analysis by fluorescence *in situ* hybridization

Fluorescence *in situ* hybridization (FISH) was performed according to the method described previously [30]. Control and detecting probes were developed from plasmids D1Z1 (1q12) and D1Z2 (1p36.3) for the chromosome 1 study and from bacterial artificial chromosomes (BACs) RP11-413 M18 (19q13) and CTZ-2571 L23 (19q13.3) for chromosome 19 study, respectively. Dual-colored probes against chromosomes 1p and 19q were used to detect chromosomal loss at these loci - a single fluorescent signal in the nucleus was interpreted as chromosomal-arm loss if two signals were detected for the control probe.

### Statistical analyses

The Student *t*-test was used to compare the mean age and KPS of patients with *IDH1* mutations. The Chi-square test was used to analyze the significance of the association between *IDH1* mutation and the following data: gender, resection rate, and *MGMT* methylation status. The overall survival was defined as the time between the first surgery and death. Survival distributions were estimated by Kaplan-Meier analysis and statistically analyzed using the log-rank test. Univariate and multivariate analysis was performed using the Cox, nonparametric proportional hazards regression model to estimate the relative risk (RR) for age, extent of resection, *IDH1* mutation status, *MGMT* status and diagnosis in group 1 and for age, extent of resection, *IDH1* mutation status, *MGMT* status, existence of 1p19q co-deletion and diagnosis in group 2, respectively. All statistical analyses were performed using StatView 5.0 (SAS Institute Inc., Cary, NC, USA).

## Results

### *IDH1* mutations in malignant gliomas

The 56 mutations of *IDH1* genes were identified in all malignant gliomas (21.1%) of the R132H type. Patients with *IDH1* mutations were significantly younger than those without IDH1 mutations (mean age, 45.5 versus 55.5 years, *P* < 0.0001). The difference in mean age was more evident in patients with GBM who had *IDH1* mutations than in those without (mean age, 43.8 versus 58.5 years, *P* = 0.004) (Table 2). *IDH1* mutations were predominantly observed in the patients with secondary GBM (8 of 12, 66.7%) but rarely in patients with primary GBM (4 of 159, *P* < 0.0001) (Table 2).

**Table 2:** **Clinical and genetic features of patients with malignant glioma with and without isocitrate dehydrogenase 1** (***IDH1***) **mutation**

		**IDH1**		
		**Mutation (+)**	**Mutation (-)**	***P*-value**
**AO**	**Cases, number**	20	10	
	**Gender**			
	Male, number	8	5	NS
	Female, number	12	5	
	**Age, mean, years**	48.3	44.4	NS
	**Karnofsky performance status, mean score, %**	94.5	89	NS
	**Surgery**			
	Total, number	16	6	NS
	Partial or biopsy, number	4	4	
	***MGMT *** **promoter**			
	Methylation (+), number	19	5	0.0155
	Methylation (-), number	1	5	
	1p 19 co-deletion, number	11	7	NS
	**Survival, median, months**	72	69	NS
**AOA**	**Cases, number**	12	14	
	**Gender**			
	Male, number	5	10	NS
	Female, number	7	4	
	**Age, mean, years**	46.4	48.7	NS
	**Karnofsky performance status, mean score, %**	97.5	96.4	NS
	**Surgery**			
	Total, number	5	8	NS
	***MGMT *** **promoter**			
	Methylation (+), number	11	8	0.0479
	Methylation (-), number	1	6	
	1p 19q co-deletion, number	7	4	NS
	**Survival, median, months**	88	65	NS
**AA**	**Cases, number**	12	28	
	**Gender**			
	Male, number	8	14	NS
	Female, number	4	14	
	**Age**,**mean**, **years**	41.7	44.3	NS
	**Karnofsky performance status, mean score, %**	90.8	78.9	NS
	**Surgery**			
	Total, number	4	4	NS
	Partial or biopsy, number	8	24	
	***MGMT *** **promoter**			
	Methylation (+), number	9	9	0.0125
	Methylation (-), number	3	19	
	**Survival, median, months**	55	25	0.0786
**GBM**	**Cases, number**	12	159	
	**Tumor occurrence**			
	Primary, number	4	155	0.0001
	Secondary, number	8	4	
	**Gender**			
	Male, number	6	99	NS
	Female, number	6	60	
	**Age, mean, years**	43.8	58.5	0.004
	**Karnofsky performance status, mean score, %**	87.5	79.7	NS
	**Surgery**			
	Total, number	3	71	NS
	Partial or biopsy, number	9	88	
	***MGMT *** **promoter**			
	Methylation (+), number	10	63	0.0032
	Methylation (-), number	2	96	
	**Survival, median, months**	20	14	0.0051

### *MGMT* promoter methylation and 1p/19q co-deletion in malignant gliomas

Of the 267 malignant glioma patients, 134 exhibited *MGMT* promoter methylation (49.4%). *MGMT* promoter methylation was considerably higher in patients with AO and AOA (80.0% and 73.1%, respectively), but relatively lower in patients with GBM (42.7%) (Table 1). Combined 1p/19q loss of heterozygosity (LOH) was noted in 60.0% AO and 42.3% AOA patients (Table 1).

### Correlation of *IDH1* mutations with *MGMT* promoter methylation and 1p/19q LOH

Gene sequence analysis showed a significant correlation of *IDH1* mutations with *MGMT* gene promoter methylation (*P* < 0.0001). *MGMT* methylation was noted in 83.3%, 75.0%, 91.7%, and 95.0% of patients with GBM, AA, AOA, and AO who had *IDH1* mutations, respectively. However, there was no significant correlation between *IDH1* mutations and LOH status of 1p/19q (Table 2).

### Survival of patients according to *IDH1* status

**Figure 1:**
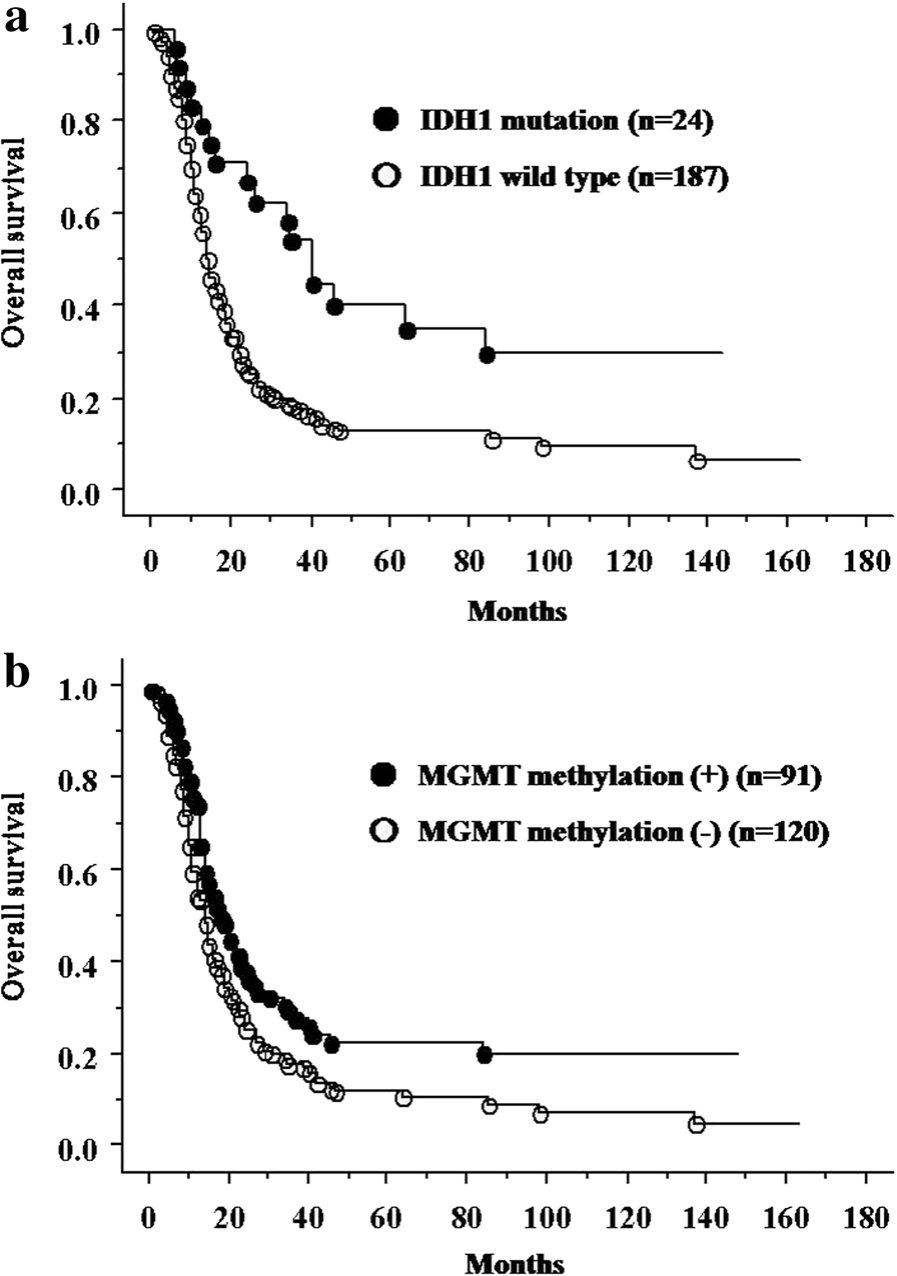
**Overall survival for anaplastic astrocytoma and glioblastoma patients. (a)** Survival of patients with glioblastoma (GBM) and anaplastic astrocytoma (AA) according to the isocitrate dehydrogenase 1 (*IDH1*) mutation status (*P* = 0.0008). **(b)** Survival of patients with GBM and AA according to the *MGMT* promoter methylation status (*P* = 0.0085).

**Figure 2:**
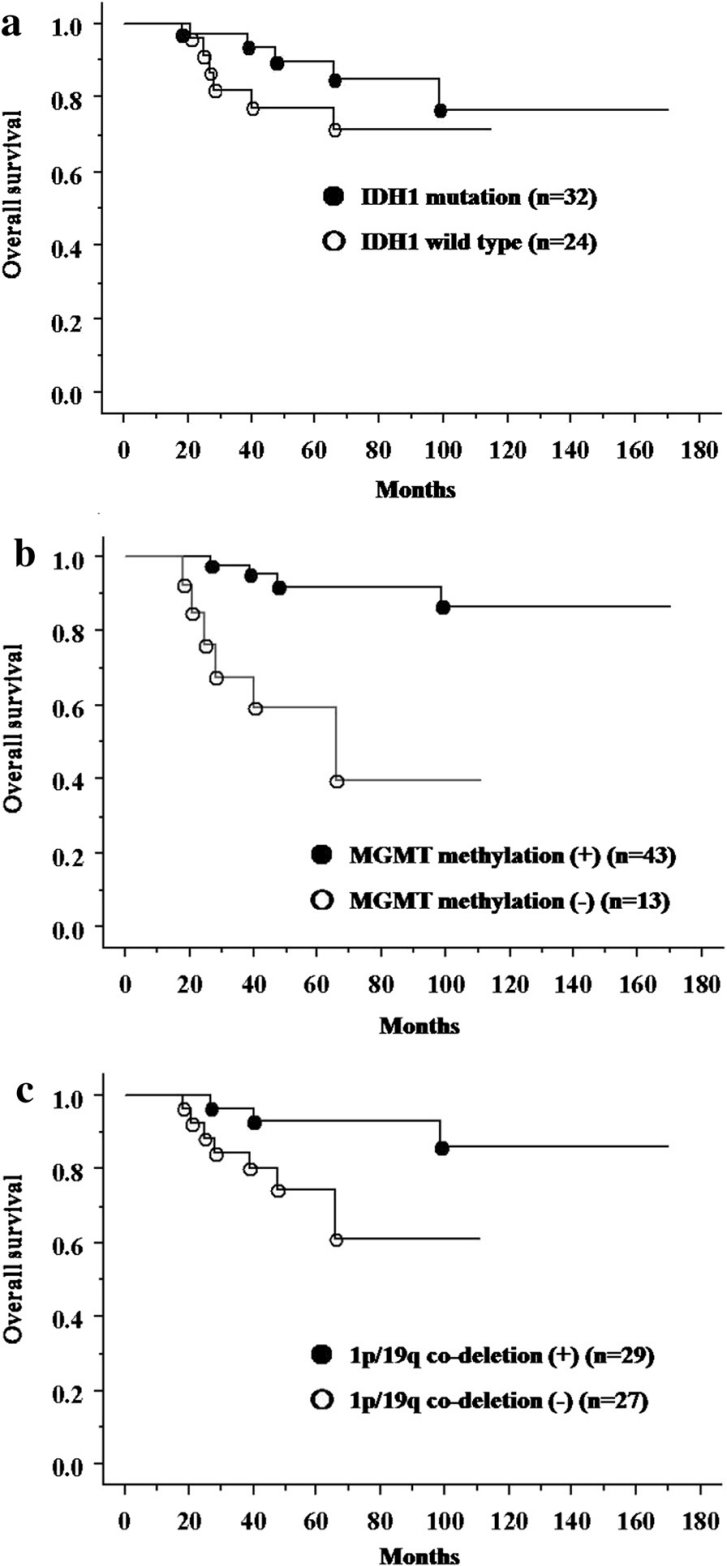
**Overall survival for anaplastic oligodendroglioma and anaplastic oligoastrocytoma patients. (a)** Survival of patients with anaplastic oligoastrocytoma (AOA) and anaplastic oligodendroglioma (AO) according to the isocitrate dehydrogenase 1 (*IDH1*) mutation (status (*P* = 0.3357). **(b)** Survival of patients with AOA and AO according to the *MGMT* promoter methylation status (*P* < 0.00001). **(c)** Survival of patients with AOA and AO according to the 1p/19q co-deletion status (*P* = 0.0228).

In group 1, patients with *IDH1* mutations had significantly longer survival time than those with wild-type *IDH1* (Figure 1a). In group 2, the survival time of patients with *IDH1* mutations was slightly longer than that of patients without *IDH1* mutations (Figure 2a).

### Survival of patients according to *MGMT* methylation status and 1p/19q co-deletion

For groups 1 and 2, patients with *MGMT* methylation had a longer survival time than those without (Figure 1b and Figure 2b). In group 2, patients with 1p/19q co-deletion had significantly better outcome than those without (Figure 2c).

### Univariate and multivariate analysis

Table 3 summarizes the significant variables. Univariately, age, gender, *IDH1* status, MGMT methylation status and histology were positively correlated with increased overall survival in group 1 (AA + GBM) (*P* < 0.05). In multivariate analysis, age, resection rate, *MGMT* status and histology were independent prognostic factor for improved overall survival in group 1 (*P* < 0.05). Also, univariate analysis showed that overall survival was significantly impacted by resection rate, *MGMT* methylation status and existence of 1p19q co-deletion in group 2 (AO + AOA) (*P* < 0.05). In multivariate analysis, age, gender and *MGMT* status were found to be independently associated with improved overall survival in group 2 (*P* < 0.05).

**Table 3:** Univariate and multivariate analysis of factors associated with survival

	**Univariate Cox regression**		**Multivariate Cox regression**			
	**HR**	**95% CI**	***P***-**value**	**HR**	**95% CI**	***P***-**value**
**Group 1** (**AA** + **GBM**)						
Age (per year)	1.023	1.014–1.033	<0.0001	1.023	1.013–1.034	<0.0001
Gender (female versus male)	1.023	1.014–1.033	<0.0001	0.810	0.590–1.112	0.1928
Resection (total resection versus non-total resection)	1.348	0.987–1.840	0.06	1.994	1.440–2.763	<0.0001
*IDH1* (mutation versus wild-type)	0.427	0.253–0.719	0.0014	0.708	0.403–1.243	0.2290
*MGMT* (methylation versus unmethylation)	0.671	0.494–0.911	0.0106	0.614	0.442–0.852	0.0035
Histology (AA versus GBM)	0.372	0.242–0.571	<0.0001	0.419	0.264–0.666	0.0002
**Group 2** (**AO** + **AOA**)						
Age (per year)	1.025	0.971–1.083	0.3672	1.094	1.003–1.193	0.0421
Gender (female versus male)	0.499	0.145–1.717	0.2703	0.156	0.027–0.890	0.0365
Resection (total resection versus non-total resection)	0.886	0.289–3.031	0.037	0.852	0.178–4.074	0.8412
*IDH1* (mutation versus wild-type)	0.563	0.172–1.848	0.3436	2.271	0.415–12.444	0.3444
*MGMT* (methylation versus unmethylation)	0.115	0.033–0.402	0.0007	0.041	0.007–0.257	0.0006
1p19q (co-deletion versus non co-deletion)	4.208	1.099–16.114	0.0359	4.720	0.685–32.526	0.1150
Histology (AO versus AOA)	0.723	0.220–2.377	0.5937	1.935	0.383–9.785	0.4247

AO, anaplastic oligodendroglioma; AOA, anaplastic oligoastrocytoma; AA, anaplastic astrocytoma; GBM, glioblastoma; HR, hazard ratio.

## Discussion

Recently, molecular markers have been increasingly used for the assessment and management of malignant glioma. Some molecular signatures are used diagnostically to help pathologists classify tumors, whereas others are used to estimate the prognosis for patients. In this study, we focused on 1p/19 co-deletion, *MGMT* promoter methylation status, and *IDH1* mutations in patients with malignant glioma.

Genetic mutations are classified into two types: driver mutations, which are involved in causing and promoting cancer, and passenger mutations, which occur concomitantly as a result of driver mutations. *IDH1* mutations have been identified as a new driver mutation by a comprehensive sequence analysis in 22 patients with GBM [13]. Interestingly, these *IDH1* mutations were associated with young patient age and secondary GBMs. This observation drew attention to diffuse astrocytoma and AA, both of which were found to carry *IDH1* mutations in the majority of cases [6,16-24]. As expected, our study also showed high frequency of *IDH1* mutations in patients with secondary GBM (66.7%) and grade 3 glioma (for example, 12 (30.0%) of 40 patients with AA, 12 (46.2%) of 26 patients with AOA, and 20 (66.7%) of 30 patients with AO), whereas the frequency was lower in patients with primary GBM (2.6%). Thus, *IDH1* mutations are thought to play an important role in the early phase of glioma development.

A relationship between good prognosis and presence of *IDH1* mutations was reported by analyzing patients with GBMs [24], AAs [6], and AOs [22]. Thus, in addition to the conventional pathological diagnosis, classification of patients on the basis of the presence or absence of *IDH1* mutations should be considered for patients with malignant glioma (GBM and AA). A study suggested that the presence of an *IDH1* mutation is a prognostic factor in AO patients [22]; however, our present study showed only slight improvement in survival of AO and AOA patients with *IDH1* mutations. Despite the absence of *IDH1* mutations, our group-2 patients had a good prognosis. In a group that includes many long survivors, determining the prognostic value becomes difficult. The difference in our results and the previous findings may be due to this reason.

*MGMT* promoter methylation has been identified in a wide range of human cancers [31]. Promoter methylation was responsible for the inactivation of this gene. *MGMT* methylation has been reported in 35% to 73% of patients with GBM [7,8,24,32-42] and 50% to 84% of patients with grade3 glioma [6,41,43]. The reported frequencies varied across studies because of the different analysis methods and conditions used in these studies. Our MS-PCR analysis showed the following frequencies of *MGMT* methylation: 42.7% (73/171), 45.0% (18/40), 73.1% (19/26), and 80.0% (24/30) for GBM, AA, AOA, and AO patients, respectively. Our study also showed significantly greater *MGMT* methylation in malignant glioma patients with *IDH1* mutations than in those without (*P* < 0.0001). Thus, these two genetic changes might have some relationship. Depending on the primers used and MS-PCR conditions, the obtained results may differ across different studies.

All *IDH1* mutations in our study involved the 132G395A mutant. G-to-A mutations are commonly found in *TP53* and *K*-*Ras* genes in patients with *MGMT* methylation [8,44]. Such common G-to-A mutations may account for the higher frequency of 132G395A mutations in the *IDH1* codon in patients with *MGMT* methylation.

Loss of 1p and 19q is thought to be the genetic hallmark of oligodendroglial tumors. The frequency of 1p/19q co-deletion was 60.0% in AO and 42.3% in AOA patients. Many studies, including three prospective randomized phase III trials, suggested that 1p/19q deletion was a powerful prognostic marker in patients with WHO grade-3 gliomas. Importantly, these studies also indicated that the prognostic power was independent of the type of adjuvant therapy, that is, radiotherapy, chemotherapy, or combined radiotherapy/chemotherapy [4-6]. We also found significantly better outcomes in Japanese patients with 1p/19q co-deletion.

**Figure 3:**
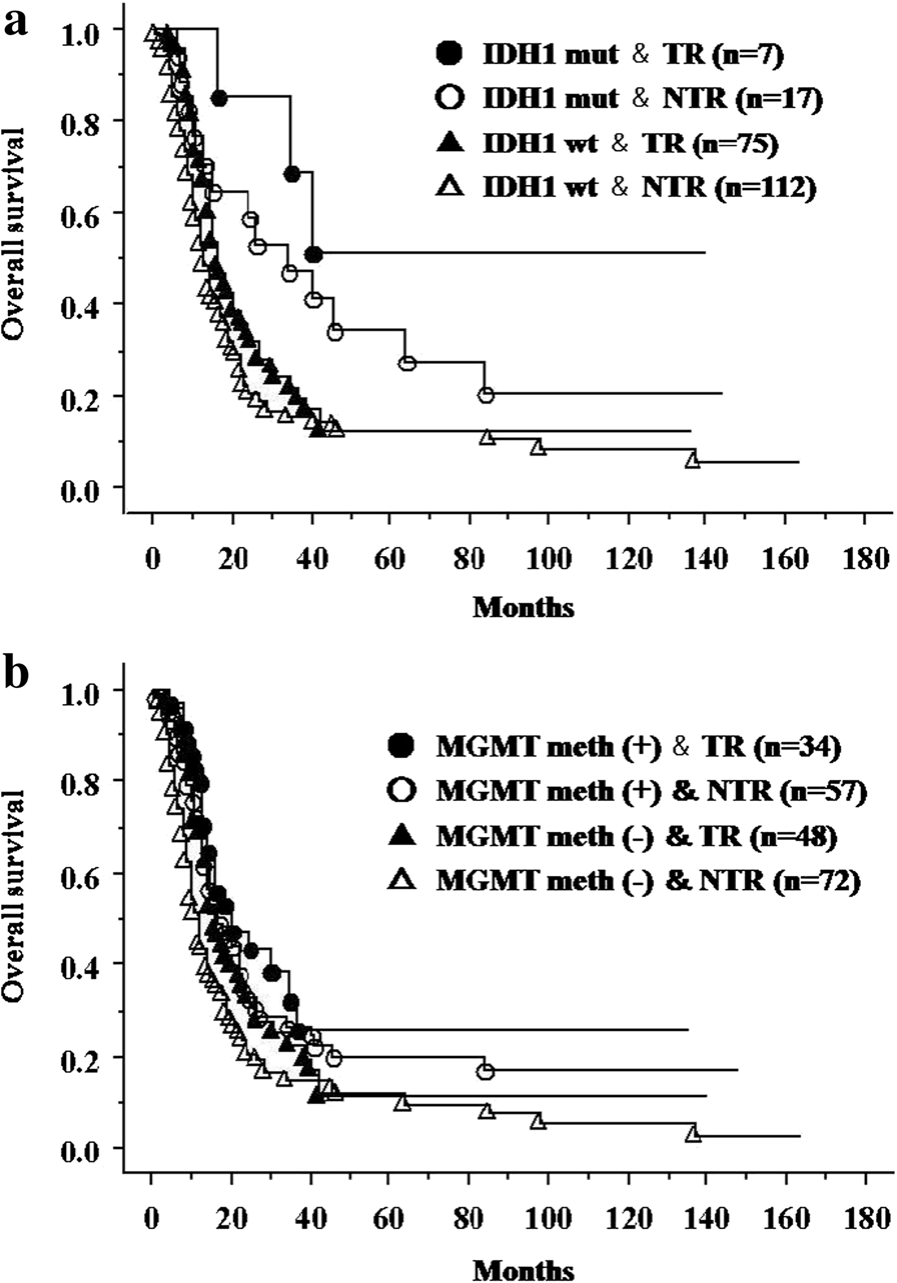
**Overall survival for anaplastic astrocytoma and glioblastoma patients according to extent of resection. (a)** Survival of patients with glioblastoma (GBM) and anaplastic astrocytoma (AA) according to the isocitrate dehydrogenase 1 (*IDH1*) mutation status and extent of resection (*P* = 0.0006). **(b)** Survival of patients with GBM and AA according to the *MGMT* methylation status and extent of resection (*P* = 0.0075).mut, mutation; wt, wild-type; meth, methylation; TR, total resection; NTR, non-total resection.

Regardless of the histological diagnosis made on the basis of the WHO classification, the surgical resection rate is considered an important prognostic factor [45,46]. Thus, we investigated the relationship between the surgical resection rate and genetic changes in *IDH1* or *MGMT* in GBM and AA patients. We obtained pre- and post-contrast magnetic resonance imaging (MRI) less than 72 hours after surgery in every case and pre-contrast and post-contrast images were compared. Enhanced areas were considered to be tumors except for obvious vessel images. The resection rate was calculated as percent change of residual tumor over preoperative T1 gadolinium (Gd) volume in all cases (100%, total removal; 95% to 5%, partial removal; below5%, biopsy). We intended to maximum resection without causing neurological morbidity. Depending on the surgical resection rate, group 1 patients were further divided into the following two subgroups: those in whom total resection was successful and those in whom total resection was not possible. In patients with *IDH1* mutations in whom total resection was not performed, the survival curves were very similar to those of patients with wild-type *IDH1* in whom total resection was performed (Figure 3). Despite the small sample size, our study suggested that the survival time of patients with *IDH1* mutations who undergo total resection is longer. If any *IDH1* mutation is considered as a marker, surgeons would be able to change their treatment strategies, including the choice of surgical procedures. Furthermore, irrespective of the *MGMT* methylation status, the prognosis of glioma patients can be improved if total resection is performed.

These findings suggest that molecular biological analyses can be used to predict the prognosis of each patient. Thus, besides the pathological diagnosis made on the basis of the existing classification system alone, developing a new classification system assessing genetic changes, such as *IDH1* mutations and the status of *MGMT* methylation and 1p/19q co-deletion, is necessary. This new classification system will allow the design of novel treatment strategies. However, information on these three genetic changes might not always be necessary. GBA and AA patients with *IDH1* mutations and *MGMT* methylation had longer survival times than those without such genetic changes. The tendency for longer survival was more marked in the subgroup with *IDH1* mutations than in those with *MGMT* methylation. Hence, for GBM or AA patients, a classification made on the basis of the presence or absence of *IDH1* mutations seems reasonable; however, that made on the basis of the *MGMT* methylation status should be discussed more carefully. The difference in the degree of association of *IDH1* mutations with prognostic factors between group 1 (GBM + AA) and group 2 (AO + AOA) patients was not clear. This could be because different numbers of patients were included in the groups. Therefore, further analyses involving a greater number of patients are necessary. Similarly, AOA and AO patients should be evaluated by taking into account the status of *MGMT* methylation and 1p/19q co-deletion, and not the *IDH1* mutation status.

## Conclusions

In summary, our study adds further support for the significant roles of *IDH1* mutations and *MGMT* methylation in the prognosis of GBM and AA patients and suggests that *IDH1* mutations might serve as a more potent prognostic factor. In contrast, *MGMT* methylation and 1p/19q co-deletion status, rather than *IDH1* mutation status, were prognostic factors in Japanese patients with AOA and AO. Furthermore, our study highlighted the importance of total resection in GBM and AA patients with *IDH1* mutations. Moreover, our study includes the largest number of Japanese patients with malignant gliomas that has been analyzed for these three markers. We believe that our findings will increase the awareness of oncologists in Japan of the value of these markers for predicting prognosis and designing appropriate therapeutic strategies for treating this highly fatal disease.

## Abbreviations

2HG: 2-hydroxyglutarate; 2OG: 2-oxoglutarate; AA: anaplastic astrocytoma; α-KG: α-ketoglutarate; AO: anaplastic oligodendroglioma; AOA: anaplastic oligoastrocytoma; CIMP: CpG island methylator phenotype; EGFR: epidermal growth factor receptor; FISH: fluorescence *in situ* hybridization; GBM: glioblastoma; G-CIMP: glioma-CpG island methylator phenotype; HIF: hypoxia-inducible factor; HR: hazard ratio; IDH1: isocitrate dehydrogenase 1; KPS: Karnofsky performance status; LOH: loss of heterozygosity; MGMT: *O*^6^-methylguanine-DNA methyltransferase; MSP: methylation-specific polymerase chain reaction; NAPD: nicotinamide adenine dinucleotide phosphate; NAPDH: nicotinamide adenine dinucleotide phosphate-oxidase; PCR: polymerase chain reaction; RR: relative risk; TMZ: temozolomide; WHO: World Health Organization.

## Competing interests

None of the authors have any financial support or conflicts of interest associated with this study.

## Authors’ contributions

YT performed all the experiments and drafted the manuscript. HN was involved in the final version of the manuscript. KM, TH and DM participated in the analyses of FISH and methylation specific PCR. HK and JK oversaw the design of the study. All authors have read and approve the final version of the manuscript.
